# Umbilical cord blood stem cells transplantation in a patient with severe progressive supranuclear palsy: a case report

**DOI:** 10.1186/s13256-021-03139-z

**Published:** 2021-11-29

**Authors:** Huiping Li, Fang Yuan, Yaming Du, Tao Pan, Wanxin Wen, Shaoxue Li, Lixin Wang, Aili Lu

**Affiliations:** Department of Neurocritical Care, The Second Affiliated Hospital of Guangzhou University of Chinese Medicine, Guangzhou, 510120 China

**Keywords:** Progressive supranuclear palsy, Umbilical cord blood, Stem cells, Case report

## Abstract

**Background:**

Progressive supranuclear palsy is a neurodegenerative condition that worsens over time. Given the lack of targeted treatments, patients with severe progressive supranuclear palsy have very low life expectancy.

**Case presentation:**

We present a case of a 61-year-old Chinese man with severe progressive supranuclear palsy and treated with umbilical cord blood stem cells transplantation. After the umbilical cord blood stem cells therapy, his neurologic symptoms stopped deteriorating, his muscle rigidity was mildly improved, and he remains alive for more than 8 years.

**Conclusions:**

Umbilical cord blood stem cells transplantation may be an alternative therapy for patients with severe progressive supranuclear palsy.

## Introduction

Progressive supranuclear palsy (PSP) is an adult-onset steady progressive neurodegenerative disease characterized by postural instability, ocular motor dysfunction, akinesia, and cognitive dysfunction [1]. PSP is associated with neurofibrillary tangles, neuronal loss, and gliosis, caused by the accumulation of microtubule-associated protein tau in the involved brain areas [2]. PSP lacks responses to dopaminergic drugs, and there are currently no targeted treatments for it. The prognosis of PSP is very poor: the median survival is only 7.3 years, and the survival of patients with severe PSP (PSP Rating Scale > 70) at 2 years is very rarely reported [3]

As a valuable donor source for allogeneic transplantation, umbilical cord blood (UCB) contains scalable pluripotent stem cells with the potential to form any human cell type in adults. Besides offering a renewable source of neural lineage [4], UCB is enriched with growth factors and cytokines that generate immunosuppressive and anti-inflammatory effects [5, 6]. Therefore, umbilical cord blood stem cells (UCBSCs) transplantation may be an alternative therapy for treating PSP. Here we report the efficacy of transplantation of allogeneic UCBSCs for a patient with severe PSP.

## Case presentation

A 61-year-old Chinese man who showed slow motion and speech at 53 years of age was admitted to our hospital in May 2012. At 54 years, gait instability, dysarthria, fine motor clumsiness, cognitive decline, and depression were observed. His difficulties in movement and postural stability then worsened gradually. At 56 years, he began to experience recurrent falls. At 58 years, he needed the aid of crutches when walking. At 59 years, he started to have incomprehensible speech, dysphagia, and double vision, and was confined to a wheelchair. Before his admission to our hospital, he had no treatments other than irregular vitamin intake. His medical and family histories were unremarkable.

**Fig. 1:**
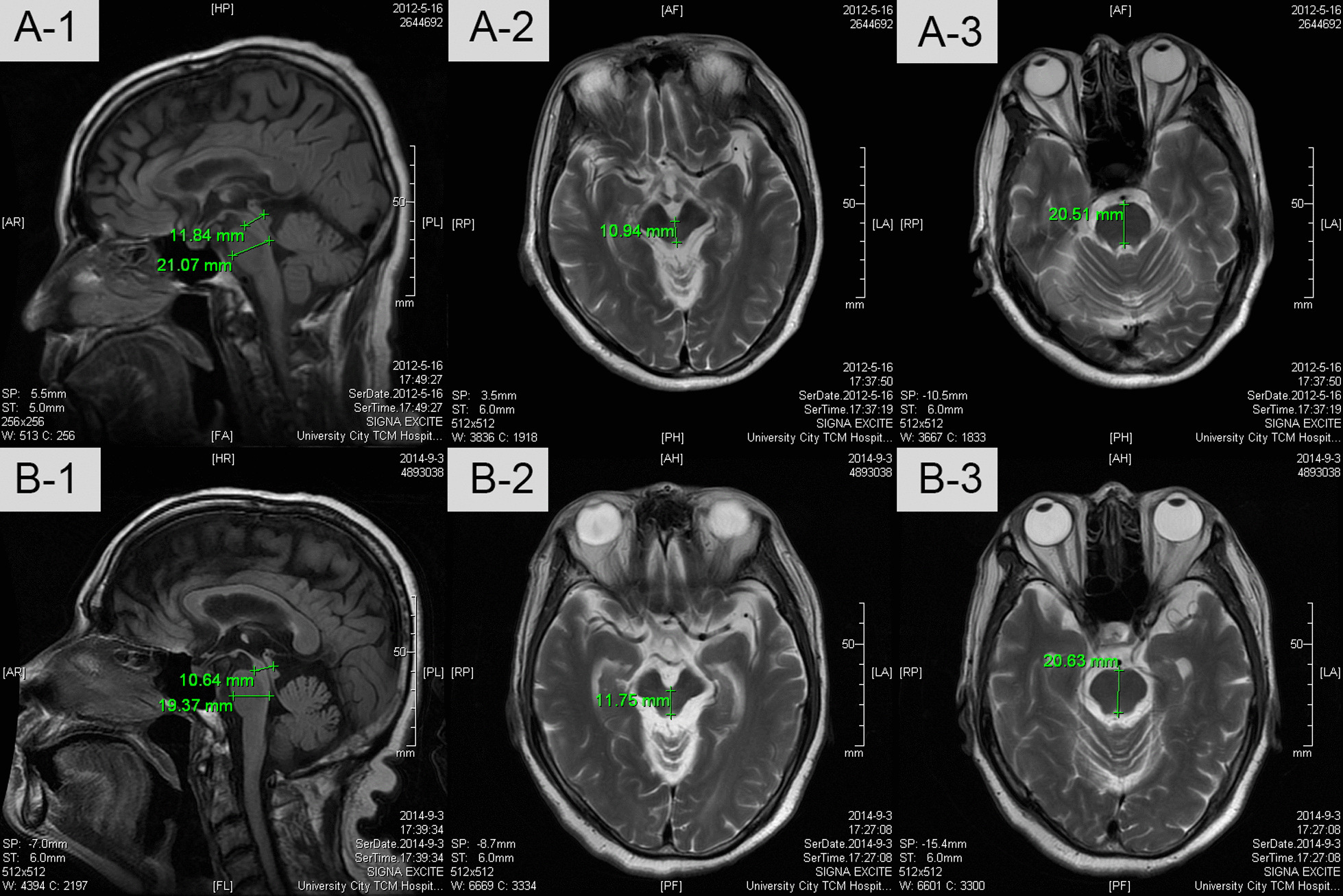
Brain MRI before and after umbilical cord blood stem cells therapy. A 1–3: MRI in May 2012. B 1–3: MRI in September 2014. The atrophy of pons and mesencephalon did not deteriorate 2 years after umbilical cord blood stem cells therapy

Neurological examination revealed cognitive decline, decreased spontaneous speech, masked face, oculomotor abnormalities, muscle rigidity, ataxia, dysarthria, and dysphagia. His cognitive impairment included apathy, marked slowing of cognition, decreased verbal fluency, and frontal release signs. His ocular movements were slow and limited in vertical direction. Muscle strength was mildly decreased in all four limbs (4/5). His limbs and axial muscles were rigid. His rapid alternating movements were clumsy and finger-to-nose and heel-to-shin movements were awkward. Resting and postural tremors in limbs were not observed. Superficial and deep sensations were intact. Deep tendon reflexes in all limbs were brisk. Sucking reflex and bilateral palmomental reflexes were present, and bilateral Babinski and Chadock signs were positive. His symptoms were not responsive to levodopa. His laboratory tests during hospitalization were unremarkable. His brain magnetic resonance imaging (MRI) scan (3.0T) suggested mild ventricle enlargement, marked atrophy of mesencephalon and cerebellum, and “Hummingbird sign” in the mesencephalon (Fig. 1).

According to clinical diagnostic criteria of PSP from the Movement Disorder Society [1], this patient was diagnosed with probable PSP with Richardson’s syndrome (prob. PSP-RS). His PSP rating scale was 73 on admission [3]. After the approval of the Ethics Committee of Guangdong Provincial Hospital of Chinese Medicine and the acquisition of patient’s informed consent, this patient received the transplantation of UCBSCs.

### Preparation of UCBSCs

**Fig. 2:**
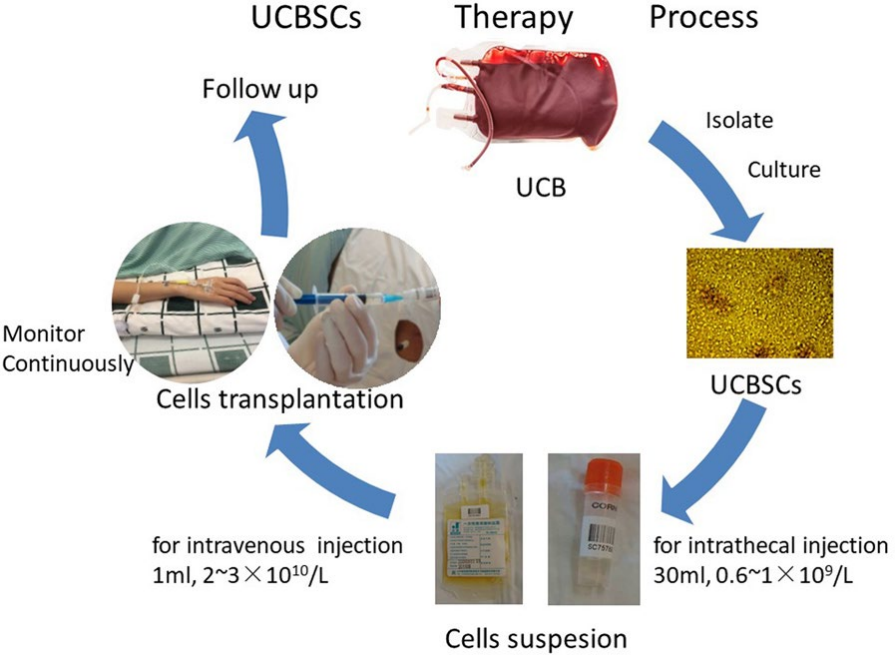
Umbilical cord blood stem cells therapy flow chart. *UCB* umbilical cord blood, *UCBSCs* umbilical cord blood stem cells

We followed previous preparation protocol of allogeneic UCBSCs (Fig. 2) [7]. The general preparation procedure is shown in Fig. 2. UCB (100 ml) was collected from healthy unrelated donors [8]. To ensure the safety of UCBSCs, pathogen detection was performed to avoid potential infection, including hepatitis B surface antigen (HBsAg) anti-hepatitis C virus (HCV), anti-human immunodeficiency virus (HIV), rapid plasma reagin (RPR), and TORCH five items. Mononuclear cells were then collected. The stem cells with a concentration of 2–3×10^10^/L were kept in 1 ml nutrient solution in a small bottle for intrathecal injection. The cells with 0.6–1.0×10^9^/L were kept in 30 ml nutrient solution in a blood bag for intravenous infusion. The stem cells were counted by whole blood analyzer syxmex100i: cell quantity of 1 unit ≥ 2–3×10^7^ cells. Cell viability was detected by COUNTSTAR Cell Counter: standard viability ≥ 90%. CD34+ cells were determined by flow cytometry (BD FACSCalibur), 1.0–2.0% CD34+ cells were required. The packed cells were placed upright in a vaccine ice box (2–8 °C). The cells were delivered to patients no more than 12 hours after preparation.

### UCBSCs therapy

The whole treatment included two intravenous infusions (30 ml, 2–3×10^7^ cells, per time) and four intrathecal injections (1 ml, 2–3×10^7^ cells, per time). The first and last injections were intravenous infusions, and the rest were intrathecal injections. The interval between every two injections was 3 days. Adverse events were closely monitored during and after the treatment.

### Outcome

**Fig. 3:**
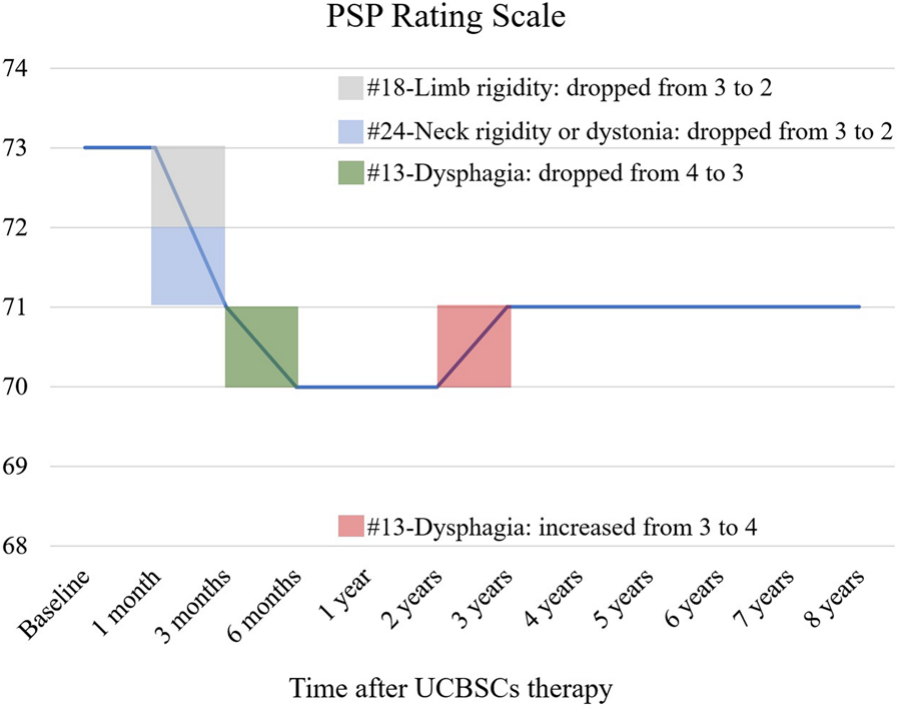
Progressive supranuclear palsy rating scale over 8 years: 73 points at baseline: #1-Withdrawal: 2; #2-Irritability: 2; #3-Dysphagia for solids: 4; #4-Using knife and fork, buttoning clothes, washing hands and face: 4; #5-Falls: 3; #6-Urinary incontinence: 3; #7-Sleep difficulty: 0; #8-Disorientation: 3; #9-Bradyphrenia: 3; #10-Emotional incontinence: 2; #11-Grasping/imitative/utilizing behavior: 3; #12-Dysarthria: 4; #13-Dysphagia: 4; #14-Voluntary upward command movement: 3; #15-Voluntary downward command movement: 1; #16-Voluntary left and right command movement: 1; #17-Eyelid dysfunction: 1; #18-Limb rigidity: 3; #19-Limb dystonia: 4; #20-Finger tapping: 2; #21-Toe tapping: 2; #22-Apraxia of hand movement: 2; #23-Tremor in any part: 0; #24-Neck rigidity or dystonia: 3; #25-Arising from chair: 3; #26-Gait: 4; #27-Postural stability: 4; #28-Sitting down: 3

This case was followed up via clinical interviews and phone calls by an experienced clinician. Three months after UCBSCs therapy, rigidity of his limbs and neck was mildly alleviated (Fig. 3). Six months after UCBSCs therapy, tube feeding was not required. The condition of this patient was stable over the next 2 years. Three years after UCBSCs therapy, the patient required tube feeding again, but the rigidity of limbs and neck was still better compared with the baseline level. All the other neurological deficits did not deteriorate until the last follow-up (through 8 years in total). No deterioration of cerebral lesions was found in his brain MRI 2 years after the treatment (Fig. 1). During the follow-up, no adverse event was observed. The progression of PSP seemed to cease after the UCBSCs therapy.

## Discussion

PSP is a progressive neurological disorder without effective treatments. Here, we presented a severe PSP case treated with UCBSCs. This patient had a PSP rating score of 73 on admission, and his score reduced by two to three points during the follow-up of 8 years. Previous studies indicated that PSP rating score in patients with PSP increases at a mean rate of 11–18 points per year, and 2-year survival was rarely reported in patients with PSP rating score over 70 [3, 9]. Our case suggested that UCBSCs transplantation might delay the progression of PSP.

Mesenchymal stem cells (MSCs) from UCB can differentiate into endothelial, epithelial, hematopoietic, and potential neural progenitor cells, and have been used to treat many neurodegenerative diseases, such as Alzheimer’s disease, Parkinson’s disease, amyotrophic lateral sclerosis, and so on [10–12]. MSCs were also demonstrated to reduce activated macrophages/microglia, attenuate reactive astrocytes, and increase white matter sparing [13–15]. In our case of severe PSP, UCBSCs transplantation delayed the disease progression, probably via the effects of neurogenesis, neuroprotection, and immunomodulation.

Bone marrow, adipose tissues, and umbilical cord blood are the most common origins of MSCs. Although MSCs are abundant in bone marrow, they are obtained via an invasive procedure that raises some ethical issues. Besides, both the proliferation and differentiation capacity of bone marrow-derived MSCs decrease with age. Previously, a case study reported that the treatment of autologous adipose tissue-derived MSCs safely delayed the progression of PSP [16]. The proliferation rate of adipose-derived MSCs is lower than UCB-derived MSCs [17]. The secretion levels of transforming growth factor-β1 (TGF-β1) and prostaglandin E2 (PGE_2_) in adipose-derived MSCs are also lower than in UCB-derived MSCs [17]. TGF-β1 exerts pleiotropic effects on the suppression of T cells and was suggested to improve the cell survival of transplanted MSCs [18]. PGE2 was also reported to enhance the therapeutic effect of MSCs in traumatic brain injury [19].

The UCB stem cells are less mature and immunogenic, and they cause less graft versus host disease (GVHD), so a perfect human leukocyte antigen (HLA) match is not required for allogeneic transplantation of UCB [20]. Our previous study also found no serious adverse events associated with UCBSCs therapy in 47 patients with cerebral palsy [8]. In this study, we chose two different routes to administer UCBSCs: intrathecal and intravenous. UCBSCs can directly reach the central nervous system by the intrathecal route, and a higher dosage of UCBSCs can be administered by the intravenous route.

The PSP rating score of this patient increased by one point in the third year after UCBSCs transplantation owing to the progressive nature of PSP. However, it remained the same until the last follow-up, and it is still lower than the baseline score. Whether repeated transplantation of UCBSCs will exert better and longer therapeutic effects needs to be explored further. This is only one case report, and randomized controlled trials are needed in the future to validate the therapeutic effect of UCBSCs transplantation in PSP and to determine the optimal dosage, administration route, and treatment duration.

## Conclusions

We presented a severe PSP case treated with UCBSCs. In this case, the UCBSCs therapy mitigated muscle rigidity, prevented the atrophy of pons and mesencephalon, and hindered the deterioration of neurologic symptoms. UCBSCs transplantation may be an alternative therapy for patients with severe PSP.

## Data Availability

Not applicable.

## Abbreviations

HCV: Hepatitis C virus
HBsAg: Hepatitis B surface antigen
HIV: Human immunodeficiency virus
PSP: Progressive supranuclear palsy
RPR: Rapid plasma reagin
RS: Richardson’s syndrome
UCB: Umbilical cord blood
UCBSCs: Umbilical cord blood stem cells
